# Comparing age-friendly city and community policies from China and the world: a systematic review

**DOI:** 10.3389/fpubh.2025.1707802

**Published:** 2026-01-09

**Authors:** Chendi Zhang, Anastasia Loukaitou-Sideris

**Affiliations:** Urban Planning, Luskin School of Public Affairs, University of California, Los Angeles, Los Angeles, CA, United States

**Keywords:** Age-Friendly Cities and Communities, age-friendly policies, older adults, China, urban planning

## Abstract

**Introduction:**

Population aging is a growing global policy and health challenge. The World Health Organization’s Age-Friendly Cities and Communities (AFCCs) framework has guided efforts to create supportive environments for older adults.

**Methods:**

This study conducts a systematic review of AFCC literature and policies in China and global contexts. The goal is to examine how China—a developing country with the world’s largest older population and unique challenges from rapid urbanization—compares to other global contexts in its response to aging.

**Results and discussion:**

The review reveals key differences of AFCC policies between China and other global regions, which include varying priorities in mobility, employment, and education policies; divergent implementation approaches; broad visions versus specific standards. Some of the differences in context-specific issues are tied to different urbanization phases. On the other hand, shared challenges include financial issues that constrain policy implementation, social exclusion, health disparities, environmental hazards and technological gaps faced by older adults, and limited policy attention to rural and suburban areas. Future directions include integrating AFCC policies into broader financial, public health, and urban agendas; supporting aging-in-place policies; developing culturally responsive strategies; addressing rural and suburban needs; incorporating technology; bridging policy and practice through implementation and monitoring; fostering participatory design of age-friendly spaces; advancing experimental research; and promoting cross-national knowledge exchanges.

## 1 Introduction

Population aging is a global trend. By 2050, the number of people 65 or older will double compared to 2022, and the percentage of this age group is projected to rise from 10% in 2022 to 16% in 2050 (1). In response to the global aging phenomenon, the World Health Organization (WHO) launched in 2007 its *Age-Friendly Cities* (AFC) initiative that includes policy recommendations and guidelines for making cities more hospitable and friendly to older adults for their physical and psychological health (2). The initiative later evolved to *Age-Friendly Cities and Communities* (AFCC) to emphasize the rural–urban continuum and include all scales of geographic settlement, not just cities (3, 4).

In response to the above initiative, regions of the Global North with high proportions of aged population, such as Europe, the U.S. and Canada, are actively engaged in AFCC programs and have developed their own AFCC policies to promote age-friendly environments (2, 4, 5). In this paper, we define AFCC policies as the strategies, plans, and regulatory guidelines developed by governments and organizations to build age-friendly cities and communities in urban and rural environments. AFCC policies typically relate to WHO’s eight domains of AFCC: housing, transportation, outdoor spaces and buildings, social participation, civic participation and employment, community support and health services, communication and information, and respect and social inclusion, and aim to support aging in place and improve older adults’ quality of life (2, 4, 6, 7).

Rapid population aging is also a phenomenon in some countries of the Global South, particularly China. China has the largest number of older adults in the world (8). Different from other Global South countries with large numbers but lower population percentages of older adults, such as India and Brazil, the percentage of people 65 or older in China reached 14.2% in 2021 and is expected to double to 29.1% by 2050 (9, 10). However, compared to many cities in Europe and North America, Chinese cities are less involved in worldwide AFCC guides and initiatives, such as *The Global Age-friendly Cities: A Guide* (AFC) (2007) and *The Global Network for Age-friendly Cities and Communities* (GNAFCC) (2018). Although Shanghai was one of the earliest member-cities that participated in the development of the 2007 AFC guide (2), only a few communities in Hong Kong Special Administrative Region (SAR) and Qiqihar City in Mainland China participated in the WHO’s GNAFCC network. People aged 60 or older in these cities and communities represent only 0.72% of the older adults in China (4). This lack of involvement in global efforts from the part of Chinese cities may be because of language gaps and cultural idiosyncrasies. GNAFCC network communications and publications are primarily in English, and occasionally in French and Spanish (4). Additionally, the general AFCC global guides may not fit well China’s cultural preferences for aged care—‘filial piety’—namely, children in a family taking care of their aging parents privately (10).

Despite its low participation in the GNAFCC, the mainland China government has considered Aging Care as one of its main goals in its national development plans since 1999 and has published a series of policies and guides on age-friendliness aiming to develop age-friendly communities for the Chinese society (10). Many of these AFCC policies and guides are effective and innovative, such as the concept and implementation of smart age-friendly homes and communities (11). Existing studies have explored China’s national aging policy—its characteristics, strengths and alignment with global AFCC frameworks (11, 12) and have examined critical factors (e.g., social isolation) influencing AFCC effectiveness in China (13, 14).

Buffel et al. (6) and Fitzgerald and Caro (15) offer comprehensive global perspectives, highlighting how AFCC frameworks have evolved across different governance systems and cultural contexts. More recently, Torku et al., (16) reviewed current AFCC studies to discover trends in age-friendly built environment research, discussing future directions for AFCC. These studies examine global and China-specific policies, providing a valuable foundation for understanding policy development, implementation, and challenges. Nevertheless, mainland China’s age-friendly policies are not included in the AFCC international reviews or comparisons (6, 15, 17). On the other hand, the conventional filial piety model can no longer fully address the aged care needs of the contemporary Chinese society (10). Chinese scholars and policymakers are referring to the more well-developed and experienced age-friendly models from other countries to enhance their age-friendly policies and guides in China (18–20). However, because of language gaps and cultural differences, few studies have covered and compared AFCC policies, guides, and related scholarly literature in China and worldwide, which has resulted in a gap in comparative knowledge about AFCC policies in China and the world (4).

Comparative analysis sheds light on existing policies and phenomena by discovering new perspectives when viewing a problem in different contexts (21). In response, this study aims to systematically collect, review, and compare the scholarly literature about AFCC policies and the AFCC policy documents discussed in the scholarly literature in China and worldwide, written in English or Chinese, to understand their differences, shared challenges, and future opportunities for effective AFCC policymaking. Thus, this study bridges a significant knowledge gap by addressing the absence of China’s AFCC policies in cross-national AFCC policy research through a bilingual systematic comparison of China’s and global AFCC policies. In addition to casting a specific light on China’s policies, the findings also advance our understanding of how different regions adapt to and shape AFCC development in diverse national settings. Specifically, the study seeks to respond to the following questions:

1. How do China’s AFCC policies compare and contrast with the equivalent policies in other countries or regions? What are the differences and shared challenges?
2. What are some future directions in the development of AFCC policies and guidelines for China and the world?

In what follows, we first give background information about the development of the AFCC initiative, followed by a discussion of aging challenges in China. Next, we present our methodology for undertaking a literature review, followed by a detailed discussion of our findings in comparing and contrasting China’s AFCC policies to those of the WHO. These findings lead us to suggest some policy implications and future directions for age-friendly policies and theories. The concluding section revisits and responds to our two research questions.

## 2 Brief overview of AFCC and aging challenges in China

### 2.1 Brief overview of AFCC and its impacts

The WHO defines Age-Friendly Cities (AFC) as places that promote active aging, ensuring older adults’ inclusion, protection, and quality of life (2, 4). Building on the *Active Aging* determinants, WHO identifies eight topic areas in the AFC checklist for cities to become more age-friendly, as mentioned earlier in the introduction section (2). To support AFC implementation, WHO launched the Global Network for Age-Friendly Cities and Communities (GNAFCC) in 2010, expanding the initial AFC members^1^ and the previous urban scope beyond cities to villages, small towns, and rural areas (4, 22, 23). The initiative evolved into Age-Friendly Cities and Communities (AFCC), emphasizing a rural–urban continuum (3, 4). AFCC promotes multisectoral action, enabling older adults to remain active contributors to families, communities, and economies (5, 24, 25). The framework is closely tied to the built environment, ensuring accessibility and inclusivity in both urban and non-urban settings.

In recent years, the WHO is leading a more up-to-date global AFCC collaboration called Decade of Healthy Aging (2020–2030), which focuses on four main areas: developing age-friendly environments, combatting ageism, improving integrated and responsive care, and providing long-term care (26). Within this broader agenda, AFCC serves as a practical, place-based mechanism for achieving the goals of Healthy Aging, translating high-level policy aspirations into concrete environmental and social interventions at local scales.

Being a comprehensive global framework designed to enhance age-friendliness in the built environment (7, 27), AFCC has significantly influenced aging policies worldwide, setting benchmarks for improving age-friendliness and raising awareness of the aging crisis (6, 27). It provides evidence-based recommendations, fosters intersectoral collaboration among policymakers, healthcare and urban planning sectors, and promotes older adult participation in decision-making (2, 4, 28, 29). Successful AFCC implementations have been documented in Ireland, Canada, New York, and Canberra (30–32, 145).

The AFCC initiative, while comprehensive and globally implemented, also faces several gaps. Language barriers limit the participation of non-English-speaking communities (4, 15). Its focus is still urban-centric, neglecting rural and suburban needs and leading to unequal implementation (28, 33). The initiative often overlooks marginalized groups such as low-income and LGBTQ+ older adults, reinforcing social inequalities (28, 34, 35). While AFCC provides general guidelines, its one-size-fits-all approach lacks local adaptability, limiting its effectiveness (36, 37). Though local actors are expected to tailor strategies, the top-down approach often fails to incorporate grassroots input, hindering innovation (7, 33).

### 2.2 Aging challenges in China

Due to its low birth rate and growing life expectancy, the Chinese government officially described China as an aging society in 1999, characterized by a significant and rapidly growing older-adult population (10). Zhang (10) outlines four phases of aging in China. The first phase (1999–2022) saw rapid aging but was mitigated by sufficient labor and resources. The second and most challenging phase (2023–2036) will witness the population peaking and transitioning into negative growth. The third phase (2037–2053) will see China becoming the world’s most severely aged country, with an expanding older-adult population and increasing social support burden. During the fourth phase (2054–2100), older-adults will stabilize at one-third of the total population due to low birth rates, forming a steady-state super-aged society (10, 38). China is currently experiencing aging challenges and will continue to face them in the coming decades.

Driven by uneven urbanization and economic disparities across provinces, the aging population in China is unevenly distributed and shaped by various factors (39, 40). Eastern coastal cities, like Shanghai, Jiangsu, and Zhejiang, have higher concentrations of older adults than the less developed cities in other regions because of their lower birth rates and appeal to retirees due to better economic development and higher degrees of urbanization. Northeastern provinces such as Liaoning experience significant aging due to economic stagnation and youth outmigration. Central China (such as Henan and Hunan) and Western China (such as Sichuan) have moderate aging populations, but face challenges in providing adequate services because they are less developed (39, 40). Rapid urbanization has transformed many rural areas into small cities, attracting older adults, but has also led to younger adults migrating to cities for work, leaving a substantial older population in rural areas, known as “left-behind” older adults. Tibet is the only province with less than 10% older adults (41). Overall, most Chinese regions face an aging crisis, with significant regional variations.

China’s aging challenges are shaped by rapid demographic shifts, regional disparities, and socio-economic transformations, making its experience distinct from many other countries. Despite some alignment with WHO’s AFC framework, China has primarily developed nation-specific policies to address its unique demographic, economic, and cultural challenges, underscoring the need for tailored, context-sensitive aging solutions.

## 3 Methodology

**Figure 1:**
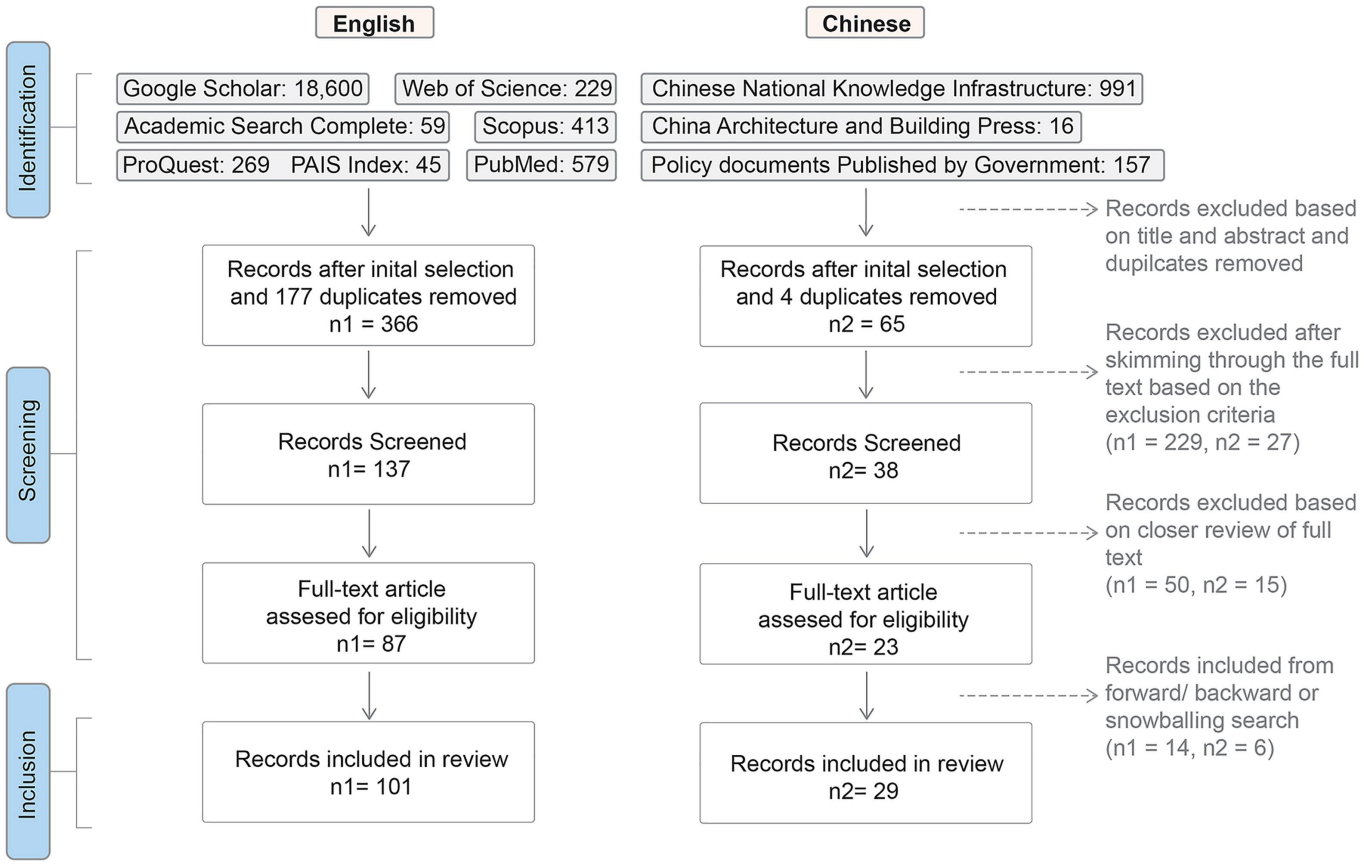
PRISMA flow diagram illustrating the systematic literature search process, document selection steps, and the numbers of documents included and excluded throughout the review (42).

We conducted a systematic search in six English databases: Google Scholar, Web of Science, Academic Search Complete, Scopus, ProQuest, PubMed, and three Chinese databases: the Chinese academic database, the Chinese public-owned architectural publisher that publishes national planning policies and guides, and official websites of the Chinese government (Figure 1). We looked for literature in three categories: (1) aging related literature using aging-related terms (Key word group A); (2) age-friendly policies and guidelines, including their review and critiques (Key word group B); and (3) age-friendly built environment topics (Key word group C) (Table 1). We undertook a title and abstract search in March 2024 and February 2025 using the combination of key word groups A, B, and C (see Appendix A).

**Table 1:** Search terms.

Key word group	Key word theme	English	Chinese
Key word group A	Aging related	age-friendly OR older adult* OR elderly* OR senior* OR age-friendly city* OR age-friendly community* OR aging* OR lifetime neighborhood* OR later life OR geriatric*	老龄化* OR 养老* OR 银发*
Key word group B	Policy and guidelines	policy* OR guideline* OR toolkit OR develop* OR initiative OR framework OR implementation OR planning OR design	政策* OR 报告*
	Review and critiques	challenge OR opportunity OR evolution OR overview OR review OR critique OR evaluation	综述 OR 探究 OR 评估
Key word group C	Built environment topics	urban planning OR urban design OR built environment	城市规划 OR 城乡规划 OR 人居环境

Asterisk (*) means the word used a search wildcard to include variations of a word root.

We followed the recommended steps of a systematic review shown in the PRISMA Flow Diagram (Figure 1). We first reviewed and excluded irrelevant documents based on title and abstract. We included: (1) studies focusing on age-friendly policies and initiatives on built environment topics rather than general aging-related research; (2) literature discussing AFCC policies; (3) peer-reviewed or official policy documents on AFCC; (4) review, critique, or comparative articles about AFCC policies; (5) documents covering national, regional, and municipal scales; and (6) documents in English or Chinese. After removing duplicates, we skimmed through the text and excluded (1) empirical studies not related to or not commenting on AFCC policies or guides; (2) studies that were not focusing on built-environment-related topics; and (3) articles published before 2007—the year when WHO first published the AFC initiative, to focus on age-friendly policies within the AFCC framework and avoid overburdening the analysis with less meaningful comparison from older and general age-friendly documents. After identifying 137 and 38 documents in English and Chinese respectively, we undertook a closer review of their full texts and excluded more ineligible documents, while including some additional documents from forward, backward, and snowballing searches. In sum, we reviewed a total of 101 English documents and 29 Chinese documents from 2007 to 2025 (Figure 1). To ensure the reliability and transparency of our review process, we also included a PRISMA checklist in Appendix B (42).

### 3.1 Analysis of policy documents

**Figure 2:**
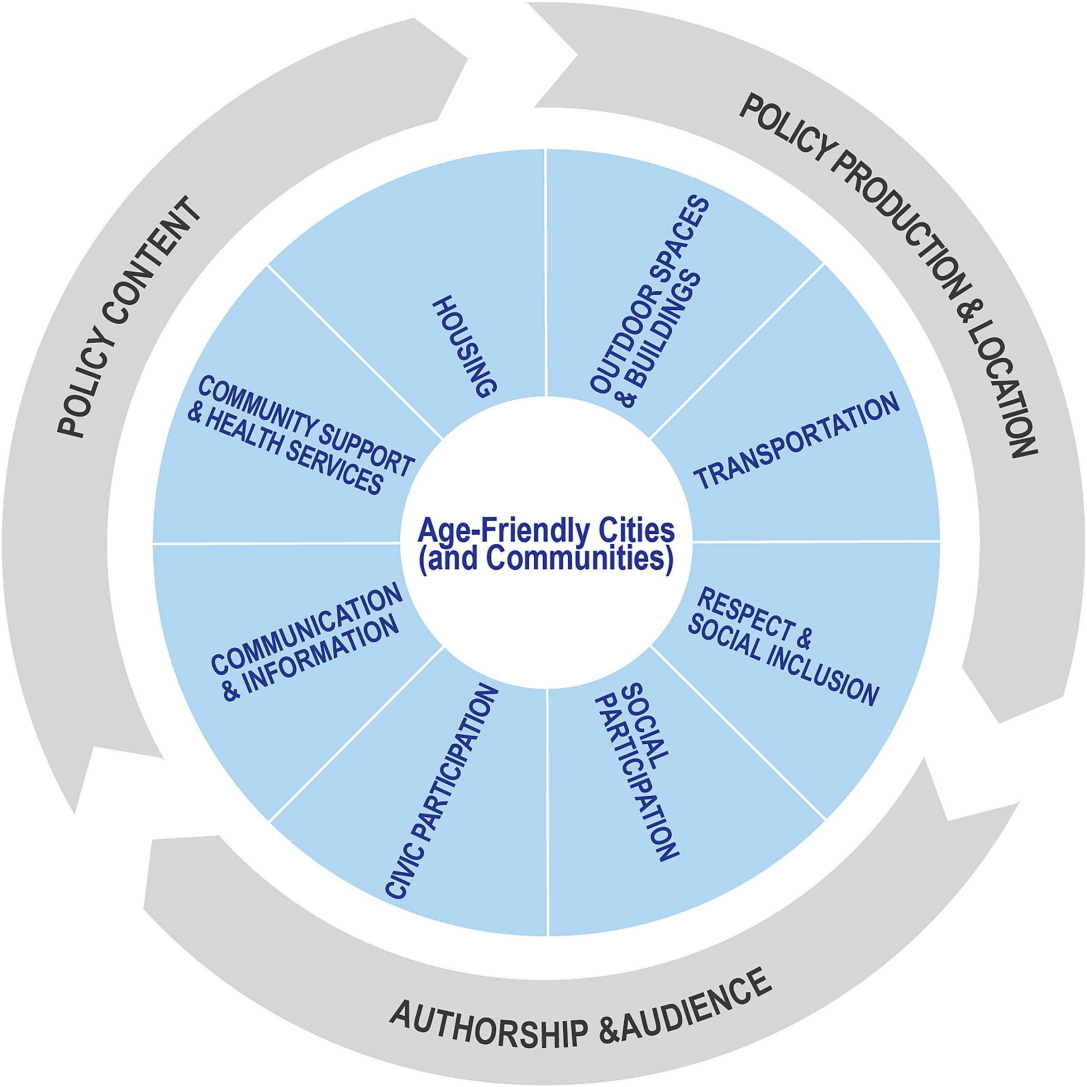
Analytical framework combining the WHO’s Age-Friendly Cities and Communities (AFCC) domains with selected themes from the Policy Document Analysis Tool (44).

We used a combined analytical framework of the WHO, Age-friendly Cities (and Communities) (defined as AFCC in the following text) guides (2, 43) and the Policy Document Analysis tool^2^ (44) to analyze policy documents. As shown in Figure 2, we examined information in each document based on the eight WHO AFCC domains across three Policy Document Analysis Tool themes: Policy Production and Location (how and where the policy was produced); Authorship and Audience (who authored the policy and for which target audience); and Policy Content. Specifically, “Policy Production and Location” included sub-codes for policy level (national, provincial, or municipal), issuing agency, and policy type (guideline, plan, or directive); “Authorship and Audience” included sub-codes for the lead authoring body, collaborating institutions, and target population (audience); and “Policy Content” was coded based on the policy strategies and action items and categorized based on the eight WHO’s AFCC domains. The coding framework and sub-codes were jointly developed by two researchers, who cross-checked the coded data through multiple rounds of discussion to achieve consensus. Conceptually, our analytical framework also parallels elements of the PICOS (Population, Intervention, Comparison, and Outcome) structure [see (45)]: the Population corresponds to the jurisdictions or policy target groups examined; the Intervention refers to AFCC policy instruments and measures; the Comparison represents the cross-national policy contrasts; and the Outcome is reflected in the thematic differences and shared challenges identified. This alignment highlights that the study captures the comparative logic of PICOS, while adapting it for qualitative policy analysis.

Policy context and policy impact were excluded from the analytical framework in this study, because the policy context across the documents was broadly similar, focusing on responding to aging populations. The cultural context, such as the notion of filial piety, is discussed only as background to explain why China’s AFCC policies differ from those in other regions, but not treated as an analytical variable, since cultural analysis is not the primary focus of this study. Moreover, evaluating policy impacts was outside the scope of this review, that had as primary goal to compare the content of AFCC policies between China and other regions. The aforementioned selected themes in the Policy Document Analysis Tool ensured a systematic and consistent qualitative evaluation of the identified policies. Our analytical framework enabled structured coding, analysis, and comparison of policy documents alongside insights from the literature.

### 3.2 Analysis of academic literature

We reviewed the academic literature to identify policy difference and shared challenges from its findings, critiques, and future directions. Specifically, to develop the thematic categories of “differences,” we compared the themes emerging from the policy contents with the findings and critiques identified in the academic literature (Table 2). Guided by the eight AFCC domains in our analytical framework, we categorized these differences according to variations in policy focus, implementation mechanisms, and contextual priorities. For the “shared challenges,” we concentrated on the critiques and future directions discussed in the reviewed literature and synthesized them into overarching thematic categories (Table 2).

**Table 2:** Differences and shared challenges synthesized from identified AFCC policy documents and literature in the review.

Themes and sub-themes		Key policy documents and literature	
		China	Worldwide regions
Differences	Encouragement of bottom-up and non-profit participation vs. embracement of institutional coordination.	*14th Five-Year Plan for the Development of the Aging Cause and the Elderly Service System (2021)* (38, 67, 68, 81, 94, 125)	*American Association of Retired Persons (AARP) Livable Communities initiative* (2005 US) *Village Movement* (2010 US) *European Thematic Network on Innovation for Age-Friendly Environments (AFE-INNOVNET)* (2014 Europe) *Ageing Well Network* (2007 Ireland) *Age-Friendly Portland Initiative* (2010 updated 2013 US) *The Active Caring Community Living Lab* (2013 Belgium) *Toronto’s Senior Strategy* (2013 Canada) *The Age-Friendly Cities and Communities-Quebec (AFC-QC) Ecological Model* (early 2010s Canada) (7, 15, 17, 23, 33, 36, 69, 70, 81, 87, 104, 111, 126, 140, 143)
	Different emphases on mobility	*TCECS 1042-2022 Technical Standard for Home-based Elderly Care Renovation of Urban Communities* (2022) *JGJ 450–2018 Standard for Design of Care Facilities for the Aged* (2018) (38, 57, 64, 74, 75, 76, 94)	*WHO − Global Age-Friendly Cities: A Guide* (2007) *WHO − Measuring the Age-Friendliness of Cities: A Guide to Using Core Indicators* (2015) *Age-Friendly Environments in Europe: A Handbook of Domains for Policy Action* (2017) *The New South Wales (NSW) Ageing Plan and Strategy* (2012, updated 2021, Australia) *Age-Friendly Portland Initiative* (2010, updated 2013, US) (136) *Age-Friendly Manchester* (2009, updated 2017, UK) (36, 66, 71, 72, 73, 77, 126)
	Employment and education vs. retirement and entertainment.	*14th Five-Year Plan for the Development of the Aging Cause and the Elderly Service System* (2021) (10, 64, 78, 77)	*Opportunity Age* (2005 UK) *A Sure Start to Later Life* (2006 UK) *Successful Ageing by the Committee on Ageing Issues* (2006 Singapore) *WHO − Global Age-Friendly Cities: A Guide* (2007) *The National Positive Ageing Strategy* (2013 Ireland) *Building a Society for All Ages* (2009 UK) *Age-Friendly NYC* (2007, updated 2017, US) *American Association of Retired Persons (AARP) Livable Communities initiative* (2005 US) *Toronto’s Senior Strategy (TSS)* (2013 Canada) *Inter-Ministerial Committee on the Ageing Population (IMC)* (1999 Singapore) *Link Age Plus* (2006 *UK*) *Opportunity Age* (2005 *UK*) (24)
	Large-scale descriptive visions vs. small-scale quantifiable standards.	*TCECS 1042-2022 Technical Standard for Home-based Elderly Care Renovation of Urban Communities (2022)* *JGJ 450–2018 Standard for Design of Care Facilities for the Aged (2018)* (18, 40, 65, 67, 68, 75, 81, 83)	*WHO − Global Age-Friendly Cities: A Guide* (2007) *The Age-Friendly Manitoba Initiative (AFMI)* (2008 Canada) *Healthy Ageing in Canada* (2006 Canada) *AdvantAge Initiative* (1999 US) *The Global Network for Age-Friendly Cities and Communities: Looking Back Over the Last Decade, Looking Forward to the Next* (2018 WHO) *Healthy Ageing in Canada* (2006 Canada) *The Age-Friendly Manitoba Initiative* (2008 Canada) (36, 80, 81)
	Neglect vs. prioritization of rural and socially vulnerable populations.	*14th Five-Year Plan for the Development of the Aging Cause and the Elderly Service System (2021)* (85, 86)	*Partnerships for Older People’s Projects* (2006 UK) *Older People Remaining at Home (OPRAH)* (2013 Ireland) *National Association of Area Agencies on Aging’s Livable Communities Initiative* (2005 US) *Ageing Well* (2010 UK) *Age-Friendly São Paulo Program – Elder-Friendly Cities Seal* (2012 Brazil) *European Thematic Network on Innovation for Age-Friendly Environments (AFE-INNOVNET)* (2014 Europe) (28, 32, 33, 36, 47, 73, 84, 104, 127, 109, 142, 145)
	Different urbanization phases.	*14th Five-Year Plan for the Development of the Aging Cause and the Elderly Service System (2021)* *National Middle-to-Long-Term Plan for Actively Addressing Population Aging* (2019) (10, 40, 57, 90, 96)	*WHO − Global Network for Age-Friendly Cities and Communities: Looking Back Over the Last Decade, Looking Forward to the Next* (2018 WHO) *Age-Friendly Manchester* (2009, updated 2017, UK) *Age-Friendly Portland Initiative* (2010, updated 2013, US) *Age-Friendly Ireland* (2009, Ireland) *Ageing Well Network* (2007, Ireland) *South Australia’s Communities for All: Our Age-Friendly Future* (2012 Australia) (32, 71, 87, 88, 102, 142, 145)
Shared challenges	Financial austerity and insufficient resources.	(10, 13, 30, 34, 88, 89, 91, 93, 94, 128, 129, 130, 92, 143, 145)	
	Differences and diverse needs among older adults.	(10, 23, 30, 35, 58, 69, 84, 95, 96, 97, 98, 116, 128, 130)	
	Social exclusion and low participation in decision-making.	(7, 58, 89, 95, 97, 99, 100, 101, 102)	
	Environmental hazards.	(7, 56, 72, 103, 104, 105, 140, 144)	
	Technology divide	(100, 106–108, 144)	
	Gaps in rural and suburban areas.	(28, 33, 36, 47, 73, 104, 109, 127)	

Our review of these documents helped us identify and collect the key AFCC policies and guides and their features in China and other regions in the world. Appendix C summarizes the identified AFCC policies, their title, authorship and audience, and content.

Following comparative policy methods (21), rather than relying on a single or consistent geographic classification (e.g., city, county, or country) as the main comparison unit, we synthesized and compared our findings based on similarities and shared challenges in policy production, audience, and contents (Appendix C). While geographic reference remains an important descriptive element, it was not used as the organizing framework for our analysis. Comparison units were not confined to the national level but also included subnational entities and regional groupings (21). Our approach prioritized policy similarities over strict geographic classification, ensuring a focus on policy approaches rather than geographic jurisdictions. We discuss our findings below.

## 4 AFCC policies worldwide and in China

### 4.1 Overview of AFCC policies globally

In the identified AFCC policies of key regions across Asia, Europe, North America, South America, and Oceania, most countries, especially countries in Europe, North America, Australia, Latin America, as well as South Korea and Indonesia in Asia, have directly used or adjusted the WHO, AFCC in their AFCC policies [Appendix C; (37, 46–50)]. Policies in Hong Kong SAR and Taiwan are also heavily influenced by and adherent to the WHO, AFCC initiative (51, 52). Singapore and Japan have adjusted their AFCC policies based on their contexts rather than fully adhering to the WHO, AFCC initiative. Singapore’s *Successful Aging by the Committee on Aging Issues* took the filial piety tradition into consideration and is defined by processes rather than goals emphasizing holistic support from the individual family, community, and national levels to facilitate successful aging (53–55). Rather than focusing on built-environment-related, social, or economic aspects, the *Health Japan 21* aims to improve public health and extend healthy life expectancy. This initiative raises awareness about lifestyle-related diseases to enhance mental and physical well-being and promote healthy lifestyles across all age groups, not just older adults (56).

### 4.2 Overview of China’s AFCC policies

Cultural differences, urbanization phases, governance structures, and the large older adult population shape the distinct characteristics of mainland China’s age-friendly policies. To address its aging crisis shaped by rapid demographic shifts, regional disparities, and socio-economic transformations (10, 39, 40), mainland China does not explicitly replicate the WHO’s guides but has developed its own AFCC policies and guides (57, 58).

China has implemented policies focusing on building age-friendliness across various scales and priorities. Key features of China’s aged care model include: (1) promoting aging-in-place as the primary mode of care; (2) supplementing institutional support with community services; and (3) integrating medical and older-adult care services to enhance health care for older adults (10, 59, 60). The *14th Five-Year Plan* outlines 11 themes to facilitate age-friendliness: achieving key indicators,^3^ improving older adult care institutions, developing the older adult health service system, enhancing medical-older adult care integration, fostering the Silver Economy,^4^ developing health-care-related technology products, promoting professional public older adult associations, promoting filial piety and respect for older adults, using smart technologies to improve life quality, building a team for policy implementation, and developing systematic older adult care evaluation measures (60). The *National Middle-to-Long-Term Plan for Actively Addressing Population Aging* (2019) discusses financial security, improving labor resources, quality aged care services, smart technologies, and inclusive social environments, emphasizing coordinated government and institutional efforts (61).

On a smaller geographic scale, China has issued specific standards to enhance age-friendliness in urban communities. The *Technical Standard for Home-based Elderly Care Renovation of Urban Communities* (2022) outlines seven domains: (1) renovation planning and general requirements; (2) urban open spaces; (3) community public environment; (4) community service facilities; (5) residential public spaces; (6) interior spaces of residential units; and (7) information services (62). Additionally, the *Standard for Design of Care Facilities for the Aged* (2018) lists requirements for aged care facilities and age-friendly buildings, including (1) site planning; (2) architectural design; (3) accessibility, indoor design, safety, hygiene, and noise control; and (4) building equipment standards (63). These standards demonstrate China’s comprehensive approach to addressing the needs of its aging population, focusing on healthcare, technology, cultural values, and economic opportunities to ensure holistic aged care across different scales (64, 65).

## 5 Comparison of policies: differences and shared challenges

**Figure 3:**
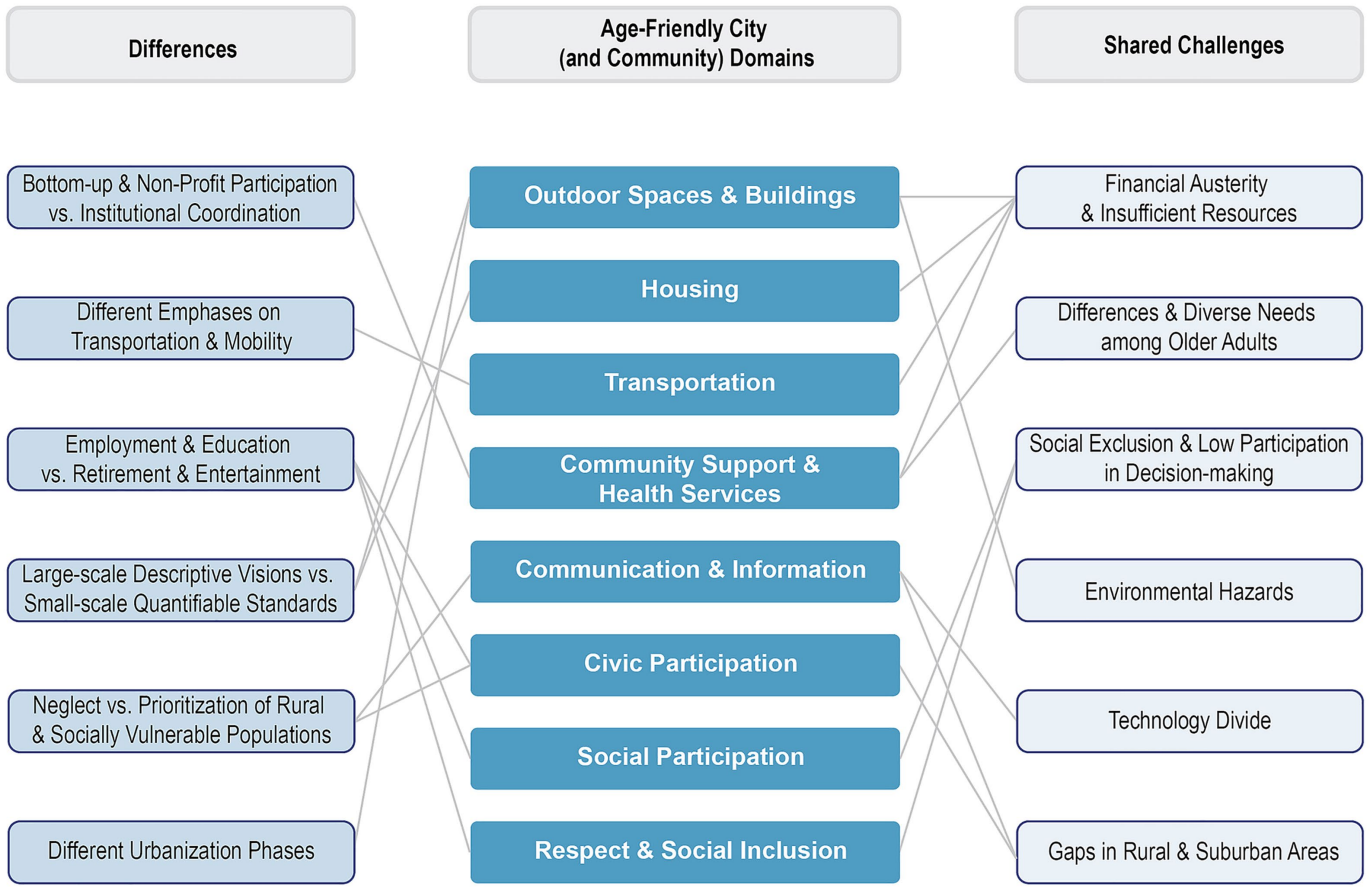
Differences and shared challenges in AFCC policies in China and worldwide across the WHO’s eight AFCC domains.

While addressing the WHO’s eight domains, mainland China’s AFCC policies place additional emphases on specific areas, such as ensuring adequate healthcare infrastructure, expanding social security systems, allocating land use for elder care, and supporting family-based elder care systems (60). These diverse aspects cover built environment, health, psychology, culture, economics, public security, and technology, greatly enriching and specifying WHO’s AFCC. In contrast, most AFCC policies in Global North countries and regions were adapted from the 2007 WHO’s AFC initiative or were developed from a country’s participation in the WHO’s GNAFCC program and usually fully match the eight topic areas of the 2007 WHO’s AFCC initiative [Appendix C; (66)]. In this section, we discuss in more detail the differences but also some shared challenges between China and other global regions in their AFCC policies, following the WHO’s eight AFCC domains (Figure 3 and Table 2). Based on our analytical framework building on WHO’s eight AFCC domains and the three out of five aspects of the Policy Analysis framework, Figure 3 was developed through thematic synthesis of both AFCC policy documents and academic literature about AFCC reviewed for this study. Figure 3 presents findings at a cross-national scale, comparing China’s national-level AFCC policies with those from other regions represented by local, national, and international initiatives. We categorized differences and shared challenges identified in the analysis and linked them to WHO’s eight AFCC domains, visually showing the connection of these differences and shared challenges to the eight domains.

### 5.1 Differences

#### 5.1.1 Bottom-up and non-profit participation vs. institutional coordination

Although many global examples of AFCC have been developed through NGO leadership and local adoption of WHO frameworks, China’s AFCC policies are formulated and implemented at the national level, reflecting its centralized governance system. This institutional difference rooted in divergent governance structures constitutes one of the key comparative findings of this study. In the domain of Community Support & Health Services, worldwide countries and regions leverage bottom-up and non-profit participation, while China focuses on institutional coordination. In terms of policy authorship and audience, several cities and countries have adopted the WHO, AFCC framework by promoting local community and nonprofit involvement to develop context-specific strategies, not only for policymakers and planners but also for community members [Table 2 and Appendix C]. Nonprofits like *Age-friendly Ireland* and *American Association of Retired Persons* (AARP) play key roles in leading community programs, advocacy, and services tailored to older adults (15). This bottom-up, community-based, and non-profit participation not only empowers local communities but also promotes context-specific innovation and adaptability. The audience of the policies is not limited to policymakers, government agencies, healthcare and social service providers, and urban planners, but targeted at community organizations, NGOs, business and private sector, and older adults, sometimes incorporating collaborations from academia and research institutions (e.g., *Age-Friendly Portland*). In contrast, China’s approach to AFCC policies embraces institutional coordination, reflecting a more top-down strategy, as seen in the “*14th Five-Year Plan for the Development of the Aging Cause and the Elderly Service System*” (60, 67). Central and local governments play a significant role in planning, funding, and implementing age-friendly policies and initiatives. Institutional coordination in China involves collaboration among various government agencies, including health, housing, and urban development departments, to ensure a unified and efficient implementation of age-friendly measures (67, 68). Although this method leverages the authority and resources of the government to achieve wide-reaching impacts and successful implementation, integrating market-led dynamics and nonprofit involvement could enhance cost-efficient, community-specific, and sustainable measures in China (57).

The contrast between encouraging bottom-up nonprofit participation in the Global North (7, 33) versus China’s systematic, top-down coordination which leverages governmental authority (10) highlights the pros and cons of two different governance models. Therefore, scholars suggest that a context-based integration of both top-down and bottom-up strategies could ensure effective and efficient implementation of age-friendly strategies and facilitate public participation (69, 70). In this case, China could leverage business and private sectors, nonprofits, and older adults’ participation to facilitate flexible, community-specific, and cost-efficient implementations to build AFCC.

#### 5.1.2 Different emphases on transportation and mobility

In the Transportation domain, countries and regions in the Global North and China have different interpretations and focuses about mobility, leading to different policy approaches (Table 2 and Appendix C). Globally, WHO-influenced policies emphasize expanding accessible, reliable, and affordable public transportation for older adults (18, 66, 71). In some Global North countries like the U.S., auto-oriented transport and low-density suburban environments have hindered public transit, making accessible transportation a priority (72, 73). Therefore, developing a more accessible public transportation system has become a mobility priority for many Global North countries. In contrast, China’s mobility focus is on small-scale, barrier-free pedestrian and cycling paths, as well as creating pedestrian-friendly environments (57, 60). While China has wide public transit coverage and a more comprehensive public transportation system with age-friendly features such as priority seating and discounted fares (74), accessibility standards were initially overlooked during the country’s rapid urbanization. However, recent years have seen a growing focus on accessible design, driven by the aging population’s needs (57, 75, 76).

#### 5.1.3 Employment and education vs. retirement and entertainment

In terms of Civic Participation, Social Participation, and Respect & Social Inclusion, worldwide countries and regions in the Global North, influenced by the WHO, AFC, emphasize the importance of continued employment and lifelong education for older adults (Table 2 and Appendix C). Policies promote flexible work arrangements, age-friendly workplaces, and anti-discrimination laws to support older adults’ engagement in the workforce. Lifelong education is also a significant focus, with initiatives aimed at providing older adults with opportunities to acquire new skills, pursue new interests, and stay intellectually engaged. This dual emphasis on employment and education helps to maintain a sense of purpose, social engagement, and financial stability among older adults, ultimately contributing to their overall well-being and quality of life (2, 24).

In contrast, China’s AFCC policies seldom mention employment or continuing education. Senior Universities^5^ are created to provide entertainment options for retired older adults rather than continued education. China’s current statutory retirement age (60 for men, 55 for women) leads to policies centered on pensions and recreational activities for retirees (64, 77, 78). However, upcoming reforms will gradually increase the retirement age, addressing demographic shifts and financial burdens (10, 79). Additionally, retirement policies that discourage older adults from working after a certain age may arouse ageism and social exclusion, and may be inappropriate for certain younger older adults, WHO, still want to work because of financial needs, for self-accomplishment, or other reasons. To cope with the rapidly increasing older population, state financial burden from pensions, and the need for inclusive working opportunities, China’s AFCC policies may need to shift from entertainment to offering more social and economic engagement opportunities for older adults to both socially and economically engage with society (60, 77).

This policy contrast has clear public-health implications. Restrictive retirement policies may inadvertently contribute to ageism, loss of purpose, and diminished mental health among older adults who wish or need to continue working. Conversely, enabling policies that support post-retirement employment, volunteerism, and lifelong learning can promote social inclusion, economic participation, and active aging. To address the challenges of a rapidly growing older population, China’s AFCC policies could further evolve from emphasizing entertainment toward expanding opportunities for older adults’ social and economic engagement, thereby advancing both individual well-being and societal sustainability.

#### 5.1.4 Large-scale descriptive visions vs. small-scale quantifiable standards

In Outdoor Spaces & Buildings and Housing domains, Global AFCC policies often adopt broad, descriptive visions based on the WHO, initiative, promoting inclusivity, accessibility, and social participation as flexible frameworks for cities to develop age-friendly environments [(2, 80, 81); Table 2 and Appendix C]. However, implementation, monitoring, and evaluation may not be guaranteed and vary from community to community (36).

By contrast, in addition to high-level visions, China’s AFC policies focus on top-down controlling requirements with small-scale, quantifiable standards (64, 81–83). These policies typically include detailed guidelines and standards for the design and construction of public infrastructure, such as specific dimensions for wheelchair ramps, tactile paving for the visually impaired, and the number of accessible public restrooms required, according to the *Technical Standard for Home-based Elderly Care Renovation of Urban Communities* (2022). Moreover, China’s *14th Five-Year Plan for the Development of the Aging Cause and the Elderly Service System* mandates minimum standards for healthcare services and Senior Universities (60). Such standards provide clear, actionable benchmarks for local governments and developers to follow, ensuring consistency and accountability in the implementation of age-friendly measures. While these benchmarks ensure effective and consistent implementation, they may lack the flexibility to adapt to local contexts.

#### 5.1.5 Neglect vs. prioritization of rural and socially vulnerable populations

In Communication & Information and Civic Participation domains, AFCC policies worldwide and in China give different emphasis on rural and socially vulnerable populations (Table 2 and Appendix C). When first developed, the WHO, AFC initiative focused primarily on middle-income older people in urban settings, while neglecting socially vulnerable and rural populations. As a result, some age-friendly policies influenced by the WHO, AFC do not prioritize the double vulnerability of older adults with low incomes, disabilities, or from minority backgrounds. They also fail to recognize different needs in rural or suburban areas (84, 109), privileging middle- and moderate-income older adults living in cities. In contrast, China’s AFCC policies place a strong emphasis on prioritizing low-income vulnerable populations and increasing awareness of improving rural age-friendliness and leaving no one behind (60). These policies often include subsidies, pension enhancements, and targeted support programs for low-income and vulnerable groups to ensure they receive adequate care and support (60, 85, 86). China’s policies explicitly show a focus on low-income populations and urban–rural disparities to reduce inequality and promote inclusive growth.

#### 5.1.6 Different urbanization phases

The above differences in policy contents and priorities mainly result from different policy contexts and urbanization phases, especially in Outdoor Spaces & Buildings. Our literature review showed that, particularly in the developed countries of the Global North, age-friendly policies focus on retrofitting and upgrading established urban environments to accommodate aging populations. These regions face the challenge of modernizing existing infrastructure, such as adapting older buildings, improving public transportation, and enhancing public space accessibility, which often involves significant investment (87, 88). Implementing these policies requires coherent efforts from local governments, private developers, and community organizations (89). Conversely, China is still experiencing rapid urbanization, characterized by new development and rural-to-urban migration, presenting opportunities to embed age-friendly principles in urban planning from the outset (40, 57). However, rapid urbanization also brings challenges, such as overcrowded cities and pressure on housing, healthcare, and social services. Additionally, the migration of younger generations to cities disrupts traditional family-based older adult care in rural areas, leaving many older adults and ‘left-behind’ children in rural regions (10, 90). This contrast underscores the different strategic focuses caused by different stages of urbanization.

### 5.2 Shared challenges

#### 5.2.1 Financial austerity and insufficient resources

One of the most significant shared challenges faced by AFCC policies and guides worldwide is financial austerity and the resulting insufficient resources manifested in Outdoor Spaces & Buildings, Housing, Transportation, and Community Support & Services [(88, 91–93); Figure 3 and Appendix C]. Many countries are grappling with tight budgets and economic constraints that limit funding for social programs, including those targeting older adults. The economic downturns and austerity measures have often led to cuts in public spending, affecting the implementation and sustainability of age-friendly initiatives (93). This financial strain makes it difficult to invest in necessary infrastructure upgrades, such as making public buildings accessible, enhancing transportation systems, and providing adequate healthcare services tailored to the needs of older adults. Additionally, there is often a lack of funding to support the development and maintenance of social programs that promote active aging and social inclusion (88, 93).

Though the context differs somewhat from that of the Global North, the Chinese government also faces the daunting task of addressing the needs of a rapidly aging population, while managing economic development and balancing budgets. Furthermore, the financial pressures on the pension system and healthcare services, exacerbated by the rapid increase of the older population, necessitate significant government expenditure, which is challenging to sustain in the long term (10). To alleviate the government’s financial and coordination burdens, China’s age-friendly policies encourage and support the private sector to facilitate aging-care business, called ‘Silver Economy’ (National Development and Reform Commission of China, 2019) (13, 94). However, the Silver Economy is at an early stage. The COVID-19 and economic downtowns have also impeded its development (13).

#### 5.2.2 Differences and diverse needs among older adults

Another major challenge for age-friendly policies and guides worldwide and in China is recognizing and adequately addressing the diverse needs of older adults in Community Support & Health Services, who vary significantly in age, socio-economic status, and health conditions (10, 84, 95–97). A one-size-fits-all approach cannot meet these differing needs, as older adults range from those newly retired and healthy in their 60s to those in advanced age with chronic conditions. Age cohorts like 60–69, 70–79, and 80+ are sometimes used, but these are not always reliable indicators of needs (98). For instance, a healthy 70-year-old may continue working, while a 60-year-old with chronic conditions may require significant care. Socio-economic disparities further affect access to healthcare and recreational opportunities, with wealthier older adults enjoying more resources (95). Additionally, LGBTQ+ older adults are often overlooked in age-friendly policies, underscoring the need for more inclusive approaches (35). However, designing such specific policies is complex and often underfunded, leading to gaps in service provision for different age groups, socio-economic statuses, and health conditions.

#### 5.2.3 Social exclusion and low participation in decision-making

In terms of Social Participation and Respect & Social Inclusion, social exclusion and low participation represent a shared critical challenge of age-friendly city policies globally [(99, 133); Figure 3 and Appendix C]. Older adults often face barriers that prevent them from fully engaging in community life, including physical limitations, lack of accessible infrastructure, technological barriers, or social stigma related to aging (97, 100, 101). These barriers can lead to a sense of exclusion and marginalization, diminishing their quality of life and overall well-being. This exclusion can lead to policies and initiatives that do not fully address the needs and preferences of older adults (102). Although the WHO’s AFC framework underscores the importance of creating inclusive environments that promote the active participation of older adults in age-friendly policy decision-making, implementing such participatory approaches requires a shift in societal attitudes and the establishment of mechanisms that facilitate meaningful engagement.

#### 5.2.4 Environmental hazards

Environmental hazards pose a significant challenge to AFCC policies globally in Outdoor Spaces & Buildings, as older adults are particularly vulnerable to air pollution, extreme weather, and climate change (103, 134). These hazards can exacerbate existing health conditions, limit mobility, and reduce the overall quality of life for seniors. For instance, poor air quality can worsen respiratory diseases, while heat waves and cold snaps can be especially dangerous for older adults with limited mobility or pre-existing health conditions. AFCC policies such as the *Age-Friendly Cities and Communities-Quebec* (AFC-QC) *Ecological Model* are increasingly recognizing the need to address these environmental risks (104, 140). This includes incorporating climate resilience into urban planning, ensuring that housing is energy-efficient and weather-resistant, and improving green spaces to reduce heat islands and improve air quality (104, 105). However, implementing such measures requires substantial investment and cross-sectoral collaboration, which can be challenging to achieve both globally and in China.

#### 5.2.5 Technology divide

In Communication & Information domain, the technology (or digital) divide, is another significant challenge for AFCC policies worldwide (Figure 3 and Table 2). As technology advances rapidly, older adults often face difficulties in keeping pace with new digital tools and platforms. Lower socio-economic statuses may impede low-income older adults’ access to technology and digital devices (106). This divide can limit their access to essential services, information, and social connections, exacerbating feelings of isolation and exclusion. For example, many services such as healthcare appointments, banking, and social interactions are increasingly conducted online, leaving those WHO, are not tech-savvy at a disadvantage. However, the assumption that older people are incapable of interacting with technologies is not completely true. Technology might not be designed for older adults but well-designed technological solutions and simplified user interfaces can be age-friendly and improve older adults’ life (107). For instance, smart applications could monitor older adults’ health condition and automatically report emergencies when necessary. Age-friendly interfaces such as large font and audio interfaces also help older adults to connect to others for more entertainment and social interactions (100, 108, 144).

#### 5.2.6 Gaps in rural and suburban areas

Although studies have pointed out the different age-friendly needs faced by residents in urban areas versus rural or suburban areas, addressing these gaps is still a shared challenge in the policymaking and implementation of AFCC policies (33, 109). Urban centers often receive more attention and resources for developing age-friendly infrastructure and services because of the concentrated larger population. Rural and suburban areas face unique challenges, such as limited access to healthcare, fewer public transportation options due to their low density, and a lack of social and recreational facilities. Older adults in these areas may experience increased isolation, limited mobility, and difficulty accessing essential services (33, 109). Addressing the gaps in rural and suburban areas is crucial for creating truly inclusive age-friendly communities both globally and in China.

## 6 Policymaking implications and future directions

Building on the shared challenges discussed previously, this section suggests policymaking implications and proposes future directions for AFCC policies, addressing the gaps and challenges identified in the literature review. Rather than treating AFCC as a static model, this discussion considers adaptations, interdisciplinary integration, and emerging trends to enhance AFCC globally.

### 6.1 Embedding AFCC policies into larger financial, public health, and urban policies

As previously discussed, one of the major shared challenges is financial austerity, both in China and many global regions. To lower the financial and resource burden, age-friendly policies should not be isolated initiatives but rather integrated into larger financial, planning, and sustainability frameworks (7, 71, 93, 110, 141). Embedding aging considerations across existing housing, transportation, healthcare, and labor policies could be a cost-efficient way to propose and implement AFCC policies (12, 111, 112). Moreover, age-friendly design overlaps with *universal design* and *intergenerational design* (113–115). Integrating AFCC policies into broader, widely adopted policy frameworks, such as general plans, zoning regulations, public space guidelines, transportation infrastructure, and housing development, can enhance their impact, facilitate successful implementation, and lower the cost of a standalone policy for older adults (6, 116). Finally, increasing older adults’ economic participation through these embedded policies is the ultimate solution to avoid agism and isolation of older adults from civic and social participation (139, 143).

### 6.2 Aging-in-place as a key trend in building AFCC

Among the policies and scholarly literature, Aging-in-Place—the ability for people to age in their home and community, is universally recognized and recommended for its economic, social, and health benefits (6, 10, 30, 49, 97, 116, 117). China’s AFCC policies also explicitly promote Aging-in-Place to encourage community-based care and alleviate the pressure on institutional systems (60). First, from the perspective of policymakers, Aging-in-Place is more cost-effective than institutional care, as it relies on individuals’ homes and local support systems, reducing the financial burden on governments and care institutions (6, 117). Second, from the perspective of older adults and their families, most prefer to remain in their homes because it allows them to maintain autonomy, preserve personal identity, and sustain emotional well-being (49, 146). Third, Aging-in-Place helps address urban–rural disparities since it does not require formal care facilities that are often lacking in rural areas (13, 97). Fourth, AIP supports more flexible, tailored approaches that acknowledge the diversity of older adult needs by age, health, income, and geography, and promotes equitable access to care and inclusion (95, 103). Overall, Aging-in-Place has become a recommended principle in building AFCC, shaping age-friendly planning toward sustainability, equity, and person-centered care.

### 6.3 Developing culture- and region-specific age-friendly policies

The WHO, AFC initiative provides comprehensive guidance and has been integrated and adopted by many regions, but its one-size-fits-all approach does not always align with local cultural and governance structures. Countries and regions that share a similar culture or context could enhance collaboration to study and tailor the AFCC model to their own context. For instance, East Asian countries, such as China and Singapore, emphasize filial piety and intergenerational responsibility, which leads to family-centered age-in-place models (118, 119). In countries or regions like China, this cultural orientation aligns with policy priorities promoting home- and community-based care but also poses challenges as traditional family support networks weaken amid urbanization and demographic change. Understanding how these cultural and structural shifts influence care patterns is essential for designing more sustainable and culturally responsive age-friendly strategies. Similarly, Latin American countries and regions with shared cultural and economic contexts may require cultural and localized frameworks that reflect community values, social networks, and resource availability. Policies should also integrate community-specific knowledge and participatory governance to improve local acceptance and effectiveness (7).

### 6.4 Addressing special needs in rural and suburban areas

The shared challenges also point out that existing AFCC policies primarily focus on urban environments, leaving rural and suburban areas underserved, but for different reasons across regions. In China, rural challenges stem from low urbanization levels, limited infrastructure, and inadequate public service systems. Many rural communities face shortages of healthcare facilities, poor transportation connectivity, and a lack of digital and social service networks, making the implementation of AFCC principles difficult without foundational development support (40, 135). By contrast, in many Global North countries, the main challenge lies in suburbanization and dispersed urban forms. Aging populations in low-density suburban and rural areas often encounter spatial and mobility barriers, such as car dependence, fragmented public transit, and poor walkability, which hinder access to services and social participation (15, 31, 109). Future research should assess the effectiveness of decentralized policy models, where local governments and NGOs lead age-friendly interventions based on regional needs.

### 6.5 Integration of technology

The literature underscores that technology holds significant potential for improving communication, mobility, and healthcare access for older adults. For example, Smart Aging measures, such as digital health monitoring, intelligent home systems, and online service platforms, have been widely implemented in pilot cities to improve care accessibility and efficiency in China (60). Early reports indicate positive outcomes in service coordination and user satisfaction, but systematic evaluations of their long-term effectiveness remain limited (120). Digital literacy gaps and usability issues remain barriers to widespread adoption (11, 106). In the long run, as the next generation of older adults becomes more technologically literate, a higher prevalence and growing familiarity with smart technologies among older adults can be anticipated (100). Policies must bridge the existing digital divide to ensure equitable access to smart aging solutions. Rather than merely introducing new technologies, future research should focus on age-friendly design principles in technology, ensuring inclusivity in user interfaces, accessibility, and data privacy (108). AI-driven personalized assistance, wearable health monitoring, and integrated smart home systems can enhance independent living and healthcare outcomes.

### 6.6 Bridging policy and practice: AFCC implementation, monitoring, and guide for older adults

Future efforts could focus on the performance of AFCC policy implementation (121, 122, 137). Establishing clear indicators and evaluation mechanisms is essential for tracking progress, identifying gaps, and ensuring accountability (22, 29). In addition, future AFCC development should emphasize designing guides not only for policymakers, but also for older adults themselves. When AFCC frameworks are communicated in accessible and user-centered formats, they can empower older adults to understand, evaluate, and participate in the transformation of their communities. Such approaches enhance autonomy, foster engagement, and improve transparency by making policy intentions and processes more visible and inclusive (22, 29, 123). Reframing AFCC tools as resources for both planners and residents can strengthen implementation by bridging the gap between policy vision and everyday lived experience.

### 6.7 Promoting creative and participatory design approaches

Current age-friendly policies and design approaches are object-focused and solutions-driven, but frame older adults as passive beneficiaries rather than active participants in shaping their environments (124). Problem-solving and top-down design approaches that lack the participation of older adults may not fully meet their needs. Architects and designers could engage with older adults to develop creative and participatory age-friendly design approaches (7, 116, 124). Alternative design approaches could be *propositional*—speculative and open-ended to function as prompts for thinking about how a space might be imagined and constructed; *relational*—building and amplifying the social connections that make up a context-specific given space; *facilitated by agency*—to enable older adults to empower themselves through the design process; and *creative and experimental*—involving art practice techniques to transform perceptions and experiences of spaces (124).

### 6.8 Longitudinal and experimental research

Built environments are dynamic and constantly evolving. Current AFCC studies are primarily cross-sectional, limiting insights into how built environments impact aging over time (112, 124). As life expectancy increases, future generations of older adults may have different needs and expectations than current ones. Longitudinal studies could examine: how urban and environmental changes impact aging experiences over decades; the effectiveness of age-friendly interventions in different socio-economic and geographic contexts; and how new aging cohorts interact with technological advancements, urban mobility, and social networks. Experimental research can explore adaptive policy models, testing pilot programs for emerging age-friendly interventions before scaling them nationally.

### 6.9 Fostering cross-national knowledge exchange in building AFCC

Cross-national knowledge exchange provides policymakers and planners with a new perspective in enhancing policy effectiveness and monitoring. Through this comparative analysis, China can learn from global models to leverage community-driven, participatory governance, nonprofit and private sector involvement to enhance local engagement and cost-efficiency policy implementation. Meanwhile, other regions can benefit from China’s top-down governance approach, which ensures national coordination, standardized policy frameworks, and large-scale implementation. In terms of policy content, China’s integration of smart technologies, rural aging prioritization, and detailed construction guides provide valuable insights for other developing nations facing similar demographic shifts and urbanization phases. Strengthening international collaboration through policy dialogues, research partnerships, and best-practice sharing will foster more inclusive, adaptable, and sustainable AFCC models worldwide (138).

## 7 Conclusion

This study systematically reviewed and compared AFCC literature and identified policy documents on AFCC policies in China and globally, highlighting their differences, shared challenges, and future directions in research and policymaking. We identified key features, strengths, and gaps of the mainstream AFCC policies influenced by the WHO, AFCC initiative and China’s own policies specific to China’s context. We also added to the literature by bringing China’s policies into the global discussion to highlight the gaps and improvement opportunities of the policies adhering to the WHO, AFCC initiative. This study fills a key knowledge gap by addressing the absence of China’s AFCC policies in cross-national AFCC research through a systematic bilingual comparison of AFCC policies in China and other global regions. It reveals contrasts between China’s and global AFCC policies in their interpretations of policy focus, scale, participation, social inclusion, and adaptability. The study also contributes to understanding the shared challenges of developing AFCC policies in China and worldwide.

In response to the first research question, in contrast to many other countries and regions that have adopted or adhere to the WHO, AFCC framework, China has developed its own set of policies based on its unique urbanization phase and cultural context, emphasizing a top-down approach with a focus on institutional coordination and specific technical standards for age-friendly environments. We find that the differences of AFCC policies and guidelines between China and other cities worldwide lie in whether they are primarily adapted from the WHO, guide or have been developed based on the country/ region’s own context; different priorities and interpretations in visions and action items such as mobility and older adult employment and education; different forms of implementation—bottom-up multi-sectoral collaboration vs. leveraging top-down efficient implementations, and broad and descriptive visions vs. specific, quantifiable standards and institutional control; and unique issues and priorities impacted by different urbanization phases. Shared challenges include financial austerity, diversity of needs among older adults, environmental hazards, social exclusion and the technological divide experienced by many older adults, and difficulties in addressing the unique needs of rural and suburban populations.

In response to the second question, we argue that future directions responding to the shared challenges should include integrating age-friendly policies into broader and existing policy frameworks for sustainability, promoting aging-in-place, developing culture- and region-specific policies, addressing the special needs of rural and suburban areas, promoting the integration of technology, bridging policy and practice through implementation and monitoring, adopting creative and participatory design approaches, pursuing longitudinal and experimental research, and fostering cross-national knowledge exchange in building AFCC.

This study fills a gap in international comparative research on AFCC policies by providing a comparative analysis of China’s approach within the global AFCC policies and AFCC framework. This cross-nation bilingual learning offers policy insights for both China and other regions to build AFCC. Nonetheless, this study has potential limitations. Some unpublished government policies and local initiatives may not have been fully captured. The language scope (English and Chinese) may have excluded relevant AFCC studies in other languages, potentially limiting a broader international comparison. Additionally, this study does not evaluate policy context and implementation, although they play a significant role in further explaining policy differences and shared challenges. Also, while different modes of governance may significantly influence AFCC policies, policy contexts, such as governance structures, are not extensively examined in this study. Future research should consider expanding data sources, evaluating policy background, policy diffusion, and governance mechanism. It should examine how different implementation strategies and administrative systems influence AFCC success, incorporating quantitative and longitudinal analyses that assess the real-world impacts of AFCC policies on health, equity, and participation outcomes. Future research should also evaluate specific AFCC policy initiatives such as the Smart Eldercare Model and Silver Economy, and should conduct case studies to further examine policy implementation gaps and performance across different geographic and governance contexts.

In conclusion, population aging is a global phenomenon with community-specific characteristics. Collaboration and exchanges among regions and countries are vital for sharing best practices, innovations, and resources to address effectively the challenges of an aging population. The needs of older adults evolve over time, particularly with urbanization and shifting societal norms, making ongoing research crucial for developing effective, evidence-based interventions. Involving older adults in research and decision-making ensures their needs are accurately addressed. Policymakers and urban planners must prioritize aging as a pressing issue, adopting comprehensive strategies and evidence-based measures to enhance the well-being and quality of life for older adults. In summary, a combination of global collaboration, local adaptation, and continuous research and participation is essential for creating truly age-friendly communities.

## Data Availability

The original contributions presented in the study are included in the article/Supplementary material, further inquiries can be directed to the corresponding author.

## Footnotes

1 The initial members of the WHO, Age-Friendly Cities (AFC) initiative in 2007 included 33 cities from 22 countries. They are Amman, Jordan; Cancún, Mexico; Dundalk, Ireland; Geneva, Switzerland; Halifax, Canada; Himeji, Japan; Islamabad, Pakistan; Istanbul, Turkey; Kingston and Montego Bay (combined), Jamaica; La Plata, Argentina; London, United Kingdom; Mayaguez, Puerto Rico; Melbourne, Australia; Melville, Australia; Mexico City, Mexico; Moscow, Russian; Federation; Nairobi, Kenya; New Delhi, India; Ponce, Puerto Rico; Portage la Prairie, Canada; Portland, Oregon, United States of America; Rio de Janeiro, Brazil; Ruhr metropolitan region, Germany; Saanich, Canada; San José, Costa Rica; Shanghai, China; Sherbrooke, Canada; Tokyo, Japan; Tripoli, Lebanon; Tuymazy, Russian Federation; Udaipur, India; Udine, Italy (2).

2 The analytical framework in Figure 2 is structured around five key aspects commonly emphasized in policy analysis literature: (1) document production and location, (2) authorship and audience, (3) policy context, (4) policy text, and (5) policy consequences. Each aspect is examined through guiding questions that help critically assess the policy’s underlying values, assumptions, and intended impacts. This approach provides a theoretical framework to analyze both the policy document and its broader policy environment, ensuring a well-informed analysis grounded in relevant literature (44).

3 The “*14th Five-Year Plan for the Development of the Aging Cause and the Elderly Service System*” sets the following key indicators to be achieved by 2025: the total number of older adult care beds will reach over 9 million; the monthly visitation rate for older adults with special difficulties will achieve 100%; the compliance rate for the construction of older adult care facilities in new urban and residential areas will be 100%; the proportion of nursing care beds in older adult care institutions will be 55%; more than 60% of secondary and higher general hospitals will have geriatric departments; the enrollment scale of older adult care-related majors in undergraduate and vocational colleges will see significant growth; the number of social workers per thousand older adults will remain above 1; each county (city, district, or banner) will have at least one university for older adults; and each county (city, district, or banner) will hold an annual “Respect for the Older Adults Month” event (60).

4 The Silver Economy includes all those economic activities, products and services designed to meet the needs of people over 60 – offered at the market, and partly supplied by senior economic actors themselves. We are referring to products and services offered to seniors, as well as those produced by them for the general market—including senior consumers (131).

5 Senior University or Elderly University, is an educational institution specifically designed to cater to the learning needs and interests of senior citizens. These universities offer courses and activities focused on hobbies, social engagement, and well-being, including subjects like health, arts, technology, literature, music, and physical exercise. The primary goal is to enrich the lives of older adults, keeping them mentally active and socially connected within their communities (132).
