# Supplementary material for: Comparing age-friendly city and community policies from China and the world: a systematic review
Source: Front Public Health. 2026 Jan 9;13:1707802. doi: 10.3389/fpubh.2025.1707802 (PMC12827672; doi:10.3389/fpubh.2025.1707802)
Supplement: Supplementary file 1 [file Supplementary_file_1.docx]

**Appendix A. Searching Syntax and the Results of Each Database.**

| Database | Searching Syntax | Results | Inclusion R1-title & abstract | After Delete Dup |
| --- | --- | --- | --- | --- |
| Web of Science | Search in Scopus  TITLE-ABS-KEY ((age-friendly OR older adult* OR elderly* OR senior* OR age-friendly city* OR age-friendly community* OR aging* OR lifetime neighborhood* OR later life OR geriatric*) AND ((policy* OR guideline OR  toolkit OR develop* OR initiative OR framework OR implementation OR planning OR design) OR (challenge OR opportunity OR evolution OR overview OR review OR critique OR evaluation)) AND (“urban planning” OR “city planning” OR “urban design” OR “built environment”))  Search in Other Databases  TITLE (((age-friendly OR older adult* OR elderly* OR senior* OR age-friendly city* OR age-friendly community* OR aging* OR lifetime neighborhood* OR later life OR geriatric*) AND ((policy* OR guideline OR  toolkit OR develop* OR initiative OR framework OR implementation OR planning OR design) OR (challenge OR opportunity OR evolution OR overview OR review OR critique OR evaluation))) AND SUBJECT (“urban planning” OR “city planning” OR “urban design” OR “built environment”) | 229 | 77 | 366 |
| Scopus |  | 413 | 125 |  |
| Google Scholar |  | 18,600 | 94 |  |
| PubMed |  | 579 | 68 |  |
| ProQuest |  | 296 | 117 |  |
| PAIS Index |  | 45 | 28 |  |
| Academic Search Complete |  | 60 | 27 |  |
| Chinese National Knowledge Infrastructure | TI = (老龄化* OR 养老* OR 银发*) AND ((政策* OR 报告*) OR (综述 OR 探究 OR 评估)) AND FIELD = (城市规划 OR 城乡规划 OR 人居环境) | 991 | 46 | 65 |
| China Architecture and Building Press | TI = (老龄化* OR 养老* OR 银发*) AND ((政策* OR 报告*) OR (综述 OR 探究 OR 评估))  **note: the subjects from the publisher are built environment related, so subjects are not included in the syntax.* | 16 | 12 |  |
| The policy database hosted by the State Council of China  https://www.gov.cn/ | 老龄化 (73) OR (老年AND 政策) (14) OR 养老AND 政策 (70)  **note: policies may not explicitly mention built environment topics, so subjects are not included in the syntax to avoid omission.* | 157 | 7 |  |

* Searches were conducted in February 2025. Asterisk (*) means the word used a search wildcard to include variations of a word root.
