# Supplementary material for: Comparing age-friendly city and community policies from China and the world: a systematic review
Source: Front Public Health. 2026 Jan 9;13:1707802. doi: 10.3389/fpubh.2025.1707802 (PMC12827672; doi:10.3389/fpubh.2025.1707802)
Supplement: Supplementary file 3 [file Supplementary_file_3.docx]

**Appendix C.** **AFCC Policies Identified in** **the Scholarly Literature and their Production, Authorship and Audience, and Policy Content.**

| **POLICY PRODUCTION AND LOCATION** | | | **AUTHORSHIP AND AUDIENCE** | | **POLICY CONTENT** | **REFERENCE** |
| --- | --- | --- | --- | --- | --- | --- |
| **Continent** | **Region** | **Key Initiative** | **Publisher/ Organizer** | **Audience** | **Summary/ Key Features** |  |
| Worldwide | Worldwide | World Health Organization - Global age-friendly cities: A guide. 2007. | World Health Organization | Targeted at governments, urban planners, public health professionals, NGOs, researchers, and community organizations to create age-friendly cities. | Guidelines for 8 topic areas: (1) outdoor spaces and buildings, (2) transportation, (3) housing, (4) social participation, (5) respect and social inclusion, (6) civic participation and employment, (7) communication and information, and (8) community support and health services. | (WHO, 2007) |
|  |  | World Health Organization. "Measuring the age-friendliness of cities: A guide to using core indicators." 2015. | World Health Organization | Municipal officials, urban planners, public health professionals, researchers, and NGOs for assessing and improving age-friendly initiatives. | Provides technical guidance on selecting and using core indicators such as equity, accessibility of the physical environment, and inclusiveness of the social environment for establishing baselines, setting goals/targets, monitoring and evaluating Age-friendly City initiatives comparably across cities and countries. | (WHO, 2015) |
|  |  | The global network for age-friendly cities and communities: Looking back over the last decade, looking forward to the next. 2018. | World Health Organization | Intended for members of the WHO Global Network, policymakers, urban planners, researchers, NGOs, and international organizations focused on aging and urban development. | The report reviews the development and achievements of the GNAFCC since 2010 and outlines strategic shifts for the next decade. GNAFCC's new directions are in line with the Sustainable Development Goals to ensure that the GNAFCC continues to foster Healthy Aging and benefit more cities and communities. | (WHO, 2018) |
| Europe | Europe | Creating age-friendly environments in Europe: a tool for local policy-makers and planners. 2016 | World Health Organization | Local policymakers, urban planners, and government officials to support age-friendly urban development in Europe | The guide aims to assist local authorities and urban planners in incorporating and implementing age-friendly policies and practices. The tool provides a comprehensive framework for creating inclusive, supportive, and accessible environments that cater to the needs of older adults. It emphasizes the importance of local action in promoting active aging and enhancing the quality of life for seniors. | (WHO, 2016) |
|  |  | Age-friendly environments in Europe: a handbook of domains for policy action (AFEE). 2017. | World Health Organization | Intended for policymakers, local authorities, and researchers focusing on age-friendly policies and community development in Europe | The handbook provides policymakers, planners, and stakeholders with a framework for designing and implementing policies that promote the well-being and active participation of older adults across Europe. It emphasizes the importance of inclusive, accessible, and supportive environments that cater to the diverse needs of the aging population. | (WHO, 2017) |
|  |  | European Thematic Network on Innovation for Age-Friendly Environments (AFE-INNOVNET). 2014. | AGE Platform Europe & European Commission | Targeted at policymakers, researchers, businesses, and NGOs working on innovative age-friendly initiatives in Europe | AFE-INNOVNET is a program in response to GCAFCC that coordinates local and regional authorities and other stakeholders – industries, research centers, universities and civil society organizations – to link up, benefit from each other’s experiences and work together to promote initiatives on age-friendly environments across the Europe. | (Rémillard-Boilard, 2018) |
|  | UK | A Sure Start to Later Life. 2006. | Social Exclusion Unit, Office of the Deputy Prime Minister (ODPM) | UK government policymakers, social service providers, and community organizations addressing social exclusion among older adults | The initiative focused on preventative services, social inclusion, and access to health and well-being support, ensuring older people could remain independent and active for ‘socially excluded’ older people. | (McGarry, 2018) |
|  |  | Link Age Plus. 2006. | Department for Work and Pensions | Local authorities, social services, and policymakers enhancing social inclusion and support services for older adults in the UK | Link Age Plus, the ‘delivery’ arm of the Sure Start to Later Life program, was a series of area-based pilot projects, designed to gather evidence around aging. It encourages partnerships between health, social care, housing, and employment services to provide seamless support for older individuals. |  |
|  |  | Opportunity Age. 2005 | Department for Work and Pensions | UK policymakers and government agencies planning long-term strategies for aging populations | Focused on employment opportunities for older adults, lifelong learning, community participation, and financial planning to promote independence. |  |
|  |  | Partnerships for Older People’s Projects. 2006. | Department of Health | Designed for local councils, health agencies, and social care providers implementing community-based support for older adults | It focuses on health promotion, social support, and preventative services, ensuring older people could maintain independence and avoid institutional care. |  |
|  |  | Building a Society for All Ages. 2009. | Department for Work and Pensions | Addressed policymakers, government agencies, and advocacy groups in the UK working towards inclusive aging policies | A successor strategy to Opportunity Age, with Ageing Well being the strategy’s delivery project. The initiative focuses on intergenerational cohesion, financial security, flexible work options, and social inclusion, addressing aging as a whole-society issue. |  |
|  |  | Ageing Well. 2010. | Department of Health | UK health professionals, policymakers, and local governments promoting healthy aging initiatives | The policy focuses on physical and mental well-being, social connectedness, and independent living, emphasizing the importance of physical activity, smoking cessation, nutrition, and quality sleep. It addresses loneliness as a major health risk, promotes accessible public transport, and highlights the challenges faced by older caregivers, particularly those providing extensive care. |  |
|  | Manchester, UK | Age-Friendly Manchester (AFM). Launched in 2009 and updated in 2017. | Manchester Health and Care Commissioning | Local government, health agencies, and community organizations in Manchester to enhance urban age-friendliness | 1) Age-friendly neighborhoods; 2) Age-friendly services; 3) Promoting age equality. Focuses on housing, transport, social inclusion, health, and economic participation for older adults. Uses co-design approaches, involving older people in planning and decision-making. Introduces local neighborhood initiatives to support aging in place and independent living. The 2017 update reinforced the city’s commitment by integrating digital inclusion, age-friendly employment strategies, and dementia-friendly communities. | (Buffel et al., 2015) |
|  |  | Manchester: A Great Place to Grow Older. 2009. | Manchester Health and Care Commissioning | Local policymakers, urban planners, and community organizations fostering age-friendly initiatives in Manchester | Set out a long-term vision to make Manchester a better place to age through investment in housing, transport, and public services. Introduced initiatives to reduce social isolation, including age-friendly cultural and recreational programs. Promoted age-inclusive employment policies, recognizing the economic contributions of older workers. | (Buffel et al., 2015) |
|  |  | Valuing Older People (VOP) partnership. 2003. | The Audience Agency, England. | UK local government, community organizations, and researchers improving aging-related policies and services. | Focused on improving older residents' quality of life through better community engagement, health programs, and public services. | (Buffel et al., 2015) |
|  | Berlin, Germany | Guidelines for Ageing Policy in Berlin. 2013. | Berlin Senate Department of Health and Social Affairs | Berlin policymakers, government agencies, and social service providers supporting aging populations. | The guidelines aim to stimulate and coordinate activities in all departments of the city government, in order to promote age-friendliness by focusing on 1) housing; 2) mobility; 3) old-age poverty. It focuses on age-friendly housing, mobility, social participation, and healthcare accessibility, ensuring older adults can live independently. | (Dale et al., 2018) |
|  | Brussels, Belgium | The Active Caring Community living lab. 2013. | Local government agencies and community organizations | Local governments, community organizations, and researchers promoting intergenerational support for aging. | The program aims to create new care concepts, services, processes and products move towards a neighborhood-organized age-in-place model of care that reinforces the autonomy of the older adult, supporting and valuing informal care. The three main projects are: 1) OPA (housing) project; 2) the Informal Neighborhood Care Networks project; 3) Case Management project. All three projects intersect with one or more of the WHO age-friendly recommended domains. It focused on integrating technology, volunteer support networks, and intergenerational programs to provide personalized and community-driven care solutions that enable older adults to remain independent. | (Smetcoren et al., 2018; Buffel et al., 2015) |
|  | Ireland | Ageing Well Network (AWN). 2007. | The Atlantic Philanthropies | Policymakers, researchers, and NGOs focusing on aging policy innovation and best practices. | The Aging Well Network filled the gap of older adult participation in age-friendly development. It had several features that were crucial to its success: 1) Inviting only senior executives to participate; 2) Creating a multi-sectoral network; 3) Including top government officials across departments and agencies; 3) Working for change within the system rather than focusing on an advocacy capacity; 4) Holding off-site retreats using the Chatham House Rule | (Shannon & O’Connor, 2015) |
|  |  | Age-friendly Ireland. 2009. | Age-friendly Ireland Shared Service Center Meath County Council, European Commission, and The Atlantic Philanthropies | Local governments, community organizations, and policymakers implementing age-friendly programs across Ireland. | The Age Friendly Ireland helps cities, counties, and towns prepare for population aging by addressing environmental, economic, and social factors affecting older adults' health and well-being. Local programs focus on walkable streets, varied housing and transportation options, essential services, and community engagement for older adults, making communities more inclusive and suitable for all ages. | (McDonald, Scharf, & Walsh, 2018; McDonald, Scharf, & Walsh, 2023; Shannon & O’Connor, 2015) |
|  |  | Ireland's Age Friendly Cities and Counties. 2010. | Age-friendly Ireland Shared Service Centre Meath County Council, European Commission, and The Atlantic Philanthropies | Irish policymakers, local governments, and advocacy groups working on regional age-friendly initiatives. | The program is part of Ireland's commitment to the WHO's GNAFCC. It is a national initiative aimed at making cities and counties more inclusive and supportive of older adults. and focuses on creating environments that promote healthy and active aging, ensuring that older adults can participate fully in community life. |  |
|  |  | Older People Remaining at Home (OPRAH). 2013 | The Ageing Well Network, Age-friendly Ireland, The Atlantic Philanthropies, and Home Instead Senior Care | Community organizations, home care providers, and policymakers supporting aging in place in Ireland. | The program promotes the age-in-place model to support older adults' successful independent living. The main recommendations in the initiative are: 1) Establish home care on a statutory basis; 2) Link the budget of the Nursing Homes Support Scheme with that available for home care packages; 3) Establish the role of the Support Coordinator at community level; 4) Conduct a comprehensive mapping of relevant resources and services available at local level; 5) Introduce a holistic needs assessment process; 6) Provide a seamless and appropriate continuum of housing options for older people; 7) Institute new and effective methods of cross-departmental and interagency working. |  |
|  |  | National Positive Ageing Strategy (NPAS) in Ireland. 2013 | Minister for Disability, Equality, Mental Health and Older People | National policymakers and advocacy groups shaping long-term aging policies in Ireland. | The National Positive Aging Strategy aims to act as a catalyst for action and innovation to promote the health, wellbeing and quality of life of people as they age in Ireland by focusing attention on issues relevant to older people across the policy development and service delivery process. Its strategies cover the 8 recommended domains of the WHO's AFC initiative. | (Shannon & O’Connor, 2015) |
|  | France | Aging Well. 2007. | The French Public Health Agency | Public health agencies, policymakers, and researchers focusing on aging and public health in France. | France's "Bien Vieillir" (Aging Well) program is a national initiative aimed at promoting healthy and active aging among its older population. Launched to address the challenges posed by an aging society, the program focuses on enhancing the quality of life for older adults through various supportive measures. It encompasses health promotion, social inclusion, and ensuring a safe and accommodating environment for seniors, which covers the WHO AFC features. | (Pennec & Le Borgne-Uguen, 2016) |
|  | Mediterranean Europe | / |  | / | Age-in-Place is the primary goal (Rodriguez-Rodriguez and Sanchez-Gonzalez 2016). | (Rodriguez-Rodriguez & Sanchez-Gonzalez, 2016) |
|  | Russia | Organization of Retired Persons “Wisdom Ripening” (ORP WR). 2010. | Organization of Retired Persons | Retired individuals, advocacy groups, and community organizations supporting older adults. | A program that participated in the GNAFCC and implements age-friendly ideas. It supports social participation, lifelong learning, and advocacy for retired individuals. | (Minnigaleeva, 2015) |
| North America | US | AdvantAge Initiative. 1999. | Visiting Nurse Service of New York (VNSNY) | U.S. local governments, aging advocates, and health organizations promoting livable communities for seniors. | The AdvantAge Initiative helps community-based organizations, foundations, and local and state governments measure their communities’ “aging friendliness” and prepare for a growing older adult population that wishes to “age-in-place.”  Four domains: 1) Basic Needs, 2) Social and Civic Engagement, 3) Optimizing Physical and Mental Health and Well Being, 4) Maximizing Independence | (Ball & Lawler, 2014; Glicksman et al., 2014; Caro & Fitzgerald, 2015; Rémillard-Boilard, 2018; Beard & Petitot, 2010) |
|  |  | Village Movement. 2010 | - Village to Village Network | Older adults, community organizers, and policymakers fostering grassroots aging-in-place support networks. | The Village Movement is an innovative grassroots movement that coordinates critical services for older adults. Villages are one of the most promising options to cope with the upcoming “Revolution Aging” dilemma. It ceated a self-managed support network offering services like transportation, social programs, and home assistance. |  |
|  |  | National Association of Area Agencies on Aging’s Livable Communities Initiative. 2005. | National Association of Area Agencies on Aging (AAAs) | U.S. regional aging agencies, local governments, and community planners. | This initiative aims to enhance the quality of life for seniors by promoting age-friendly policies, programs, and services that enable them to live independently and actively participate in their communities. The key features are: 1) Collaborate Across Traditional and Nontraditional Sectors; 2) Celebrate Racial and Ethnic Diversity; 3) Build Individual Relationships; 4) Honor Your Unique Local Community; 5) Revere Older Adults; 6) Embrace Longevity as an Opportunity; 7) Tackle the Social Factors that Determine Community Wellness; 8) Seize Opportunities to Infuse Age in Everything; 9) Send the Right Messages; 10) Leverage Local Dollars for Livability. |  |
|  |  | Building Healthy Communities for Active Aging (BHCAA) Award Program. 2006. | U.S. Environmental Protection Agency | U.S. policymakers, local governments, and community organizations promoting active aging. | It awards cities that implemented policies promoting walkability, accessibility, and environmental sustainability for older adults. This award program was created to recognize and promote communities that are taking steps to improve the health and well-being of older adults through smart growth and active aging strategies. The program aims to highlight successful initiatives that integrate environmental protection, land use planning, and public health, fostering communities where older adults can live actively and independently. |  |
|  |  | American Association of Retired Persons (AARP) Livable Communities initiative. 2000, updated 2005. | American Association of Retired Persons (AARP) | U.S. policymakers, urban planners, and community organizations enhancing livability for aging populations. | The AARP Livable Communities initiative supports the efforts of neighborhoods, towns, cities, counties and entire states to become more livable and age-friendly for people of all ages. The main domains are: housing, transportation, public spaces, socialization, shopping and health care services, employment, community engagement and empowerment. |  |
|  | New York, US | Age-friendly NYC. Launched in 2007 and updated in 2017. | the Office of the Mayor, the New York City Council, and the New York Academy of Medicine | New York City government, local organizations, and researchers. | This initiative aims to enhance the quality of life for the city’s aging population by addressing various aspects of urban living, including housing, transportation, social participation, and healthcare. | (Goldman et al., 2016) |
|  | Portland, US | Age-friendly Portland Initiative. 2010, updated in 2013. | The Institute on Aging (IOA) at Portland State University, in partnership with local government, community organizations, and businesses. | Portland policymakers, researchers, and local organizations advancing age-friendly urban planning. | The Age-Friendly Portland Initiative is a comprehensive effort to make Portland, Oregon, more inclusive, supportive, and accessible for older adults. Launched as part of the city's commitment to the World Health Organization’s Global Network of Age-Friendly Cities and Communities, the initiative aims to enhance the quality of life for older residents through strategic planning, community engagement, and multi-sector collaboration. | (Delatorre & Neal, 2015; Neal et al., 2014) |
|  | Philadelphia, US | Age-friendly Philadelphia. 2009. | Philadelphia Corporation for Aging (PCA). | Philadelphia city officials, community organizations, and researchers. | PCA’s mission is to improve the quality of life for older Philadelphians and people with disabilities and to assist them in achieving their maximum level of health, independence and productivity. Domains are adapted from the WHO's AFC guide. | (Glicksman & Ring, 2015; Glicksman et al., 2014) |
|  | Atlanta, US | Lifelong Community Initiative. 2007. | Atlanta Regional Commission. | Regional policymakers, community planners, and communities. | This program of the Atlanta Regional Commission pays special attention to the needs of seniors as it works to improve community design through city-wide ordinances that support better walking and transportation alternatives and healthy housing for seniors. | (Keyes et al., 2015) |
|  | Canada | Healthy Ageing in Canada. 2006. | Public Health Agency of Canada (PHAC). | Canadian public health agencies, policymakers, and researchers. | "Healthy Aging in Canada" is a comprehensive framework developed to promote the well-being and quality of life for older adults across the country. This initiative focuses on enabling seniors to lead healthy, active, and independent lives by addressing key areas such as health promotion, social inclusion, and supportive environments. It is a collaborative effort involving federal, provincial, and territorial governments, as well as community organizations and stakeholders. | (Nykiforuk et al., 2019; Plouffe & Kalache, 2011) |
|  | Toronto, Canada | Toronto's Senior Strategy (TSS). 2013. | City of Toronto. Supported by Toronto Seniors’ Forum, Toronto Council on Aging, local community organizations, and academic institutions. | Toronto government agencies, advocacy groups, and researchers addressing aging policy. | The first and second Toronto Seniors Strategies advanced key city initiatives that supported the quality of life, social participation, access to services, and well-being of seniors in Toronto. The key features are: 1) Integrated city senior housing and services entity; 2) Health; 3) Housing; 4) Transportation; 5) Employment and income; 6) Access to information. | (Joy, 2018) |
|  | Quebec, Canada | The Age-Friendly Cities and Communities-Quebec (AFC-QC) Ecological Model. Early 2010s. | Quebec Government. | Quebec government agencies, urban planners, and community organizations. | The Age-Friendly Cities and Communities-Quebec (AFC-QC) Ecological Model is a framework designed to guide the development of age-friendly environments in Quebec. This model emphasizes the interaction between older adults and their environments, considering various ecological levels that influence aging, such as individual, community, and societal factors. | (Garon et al., 2015; Garon et al., 2016) |
|  | Manitoba, Canada | The Age-Friendly Manitoba Initiative (AFMI). 2008. | Government of Manitoba (Manitoba Seniors and Healthy Aging Secretariat). | Manitoba policymakers, local governments, and aging service providers. | The Age-Friendly Manitoba initiative is a comprehensive multifaceted approach that contributes to the health and well-being of its citizens. Viewing community living through an Age-Friendly lens helps to improve quality of life for all and contributes to a respectful social environment. It is built on the World Health Organization’s (WHO) healthy aging framework that assesses eight key dimensions of community life where opportunities are optimized for health, participation and security. | (Menec et al., 2015; Menec, 2022) |
| Oceania | Australia | South Australia’s Communities for All: Our Age-Friendly Future, Age-friendly South Australia Guidelines for State Government. 2012. | Government of South Australia. | South Australian state policymakers and local governments promoting age-friendly policies. | This is a strategic initiative aimed at creating age-friendly environments across the state. It provides guidelines for local governments to improve transport, housing, health services, and social participation. It emphasized collaboration between state and local governments to implement age-friendly policies. It seeks to enhance the quality of life for older adults by fostering inclusive, supportive, and accessible communities. It is part of South Australia's commitment to the World Health Organization’s Global Network of Age-Friendly Cities and Communities and focuses on addressing key areas such as health, social inclusion, housing, and community engagement. | (Kendig et al., 2014; Kendig et al., 2018; Brasher & Winterton, 2016) |
|  |  | Canberra Plan - An Age-Friendly City Project  Age-friendly City Plan. 2010, updated in 2020. | Australian Capital Territory, Canberra. | Canberra policymakers, urban planners, and community organizations. | The plan embraces diversity for all ages in a city. The key features are: Involved; Connected and valued; Safe; Secure and free from abuse; Information; Service and supports. It focused on accessible public transport, age-friendly housing, public spaces, healthcare access, and digital inclusion. The 2020 update expanded the scope to address climate resilience, technology integration, and social inclusion for seniors. |  |
|  |  | The New South Wales (NSW) ageing plan and strategy. 2012, updated in 2021. | New South Wales (NSW) government. | NSW policymakers, local councils, and service providers addressing aging challenges. | The strategy aims to create a more inclusive, supportive, and age-friendly society, enabling older adults to lead healthy, active, and fulfilling lives. The strategy focuses on several key areas, including health and well-being, social inclusion, economic participation, housing, and community support. The 2021 version also focused on elder abuse prevention, mental health support, and digital inclusion. |  |
| Asia | Singapore | Inter-Ministerial Committee on the Ageing Population (IMC). 1999. | Inter-Ministerial Committee on the Ageing Population (IMC) | Singapore’s government agencies coordinating aging policies. | Six key areas – 1) Financial security, 2) Employment and employability, 3) Housing and land use policies, 4) Health care, 5) Social integration of the elderly, and 6) Cohesion and conflict in an aging society (intergenerational bonding). It emphasized the importance of community-based aging solutions and lifelong learning. | (Yew, 2020; Chong et al., 2015) |
|  |  | Successful Ageing by the Committee on Ageing Issues (CAI) in 2006. | The Committee on Ageing Issues (CAI) | Singaporean policymakers and researchers. | The underlying philosophy of “Successful Aging” is holistic support from all levels: the “individual level,” the “family level,” the “community level,” and the “national level.” The initiative emphasizes the financial security, mobility, housing, and family and intergenerational bonding to facilitate successful aging in Singapore. |  |
|  | Japan | Health Japan 21. 2013. | National Institute of Health and Nutrition, Japan | Japan’s health policymakers, researchers, and public health organizations promoting aging well. | Health Japan 21 is a national health promotion initiative launched by the Japanese government to improve public health and extend healthy life expectancy. The initiative aims to address lifestyle-related diseases, enhance mental and physical well-being, and promote healthy lifestyles across all age groups. | (Koohsari et al., 2018) |
|  | South Korea | Adopted and applied the WHO AFC guides to major cities | / | City governments, urban planners, and policymakers. | Adopted and applied the WHO AFC guides to major cities (Lee and Kim 2020). | (Lee & Kim, 2020). |
|  | Indonesia | Embedded in other social welfare and public policies. | / | City governments, urban planners, and policymakers. | Key age-friendly measures and regulations identified in the literature are:  1) 2009, emphasized on social welfare; 2) 2013, conducted an AFC assessment; 3) 2015 Balikpapan Regulation Senior and Welfare Malang Regulation on Senior Welfare; -4)2016 Jakarta Regulation providing free Transjakarta (rapid bus transit system) services for specific groups, including seniors (Suriastini, Buffardi, and Fauzan 2019). | (Suriastini et al., 2019) |
|  | China (mainland) | TCECS 1042-2022 Technical Standard for Home-based Elderly Care Renovation of Urban Communities. 2022. | China Association for Engineering Construction Standardization (CECS) | Chinese policymakers, architects, urban planners, and construction professionals. | The technical standard aims to improving the living environment for older adults to meet their physical, mental, and behavioral needs and providing a guide to evaluate and monitor the age-friendly urban community renewal and renovation.  The key sections are: 1) renovation planning and general requirements; 2) urban open spaces surrounding the community; 3) community public environment 4) community service facilities; 5) residential public spaces; 6) interior spaces of residential units; and 7) information services. | (Hu, 2023; Shi et al., 2022) |
|  |  | 14th Five-Year Plan for the Development of the Aging Cause and the Elderly Service System. 2021. | The State Council of the PR China | Chinese government agencies, policymakers, urban planners, researchers, and local governments. | The goals and action items of the plan are: 1) Achieving the main indicators listed in the 14th Five-Year Plan for the Development of the Aging Cause and Elderly Care Service System; 2) Improving public elderly-care institutions and services; 3) Developing the elderly health service system; 4) Enhancing the integration of medical and elderly care Services; 5)Planning and developing key regions for the Silver Economy; 6) Studies and manufactures of technologies and applications for older adults; 7) Normalizing and monitoring the establishments of public elderly associations; 8) Inheritance and innovation of Chinese Filial Piety and elderly respect culture; 9) Smart technologies to improve older adults’ life quality; 10) Building a team for age-friendly policies implementation and elderly care services; 11) Systematic elderly care service measures. | (Hu, 2023; Shi et al., 2022; Huo, 2023) |
|  |  | National Middle-to-Long-Term Plan for Actively Addressing Population Aging. 2019. | National Development and Reform Commission (NDRC) | Chinese government agencies, policymakers, urban planners, researchers, and local governments. | The plan aims to provide for middle-to-long-term age-friendly strategies to cope with the growing aging population in China.  The key objectives and action items are 1) financial security, 2) more qualified labor resources to compensate for the declining number of labor resources, 3) quality aged care services and resources, 4) (smart) technologies, and 5) age-friendly inclusive social environments. | (Shi et al., 2022; Yuan & Jin, 2020; Li and Yue, 2020; Zhang, 2019; Li & Zheng, 2019; Lu, 2019) |
|  |  | JGJ 450-2018 Standard for Design of Care Facilities for the Aged. 2018. | Construction engineering industry construction standards (JGJ)） | Architects, urban planners, and construction professionals. | The standard aims to provide a technical guide to build age-friendly facilities and housing.  The key sections are 1) site and planning; 2) architectural design; 3) special requirements for accessibility design, indoor design, safety, hygiene, and noise control; and 4) building equipment standards. | (Zhang, 2019; Chen & Zhang, 2018; Zhou, 2016; Zhang et al., 2015; Li & Zheng, 2019; Lu, 2019) |
|  | Hong Kong Special Administrative Region, China | Age-friendly Hong Kong Streering Committe (WHO AFCC 2007). 2008. | Age-friendly Hong Kong Steering Committee | Hong Kong government agencies and advocacy groups. | The initiative was established to guide and promote the development of age-friendly policies and practices across Hong Kong. This committee works towards creating an inclusive, supportive, and accessible environment for older adults, ensuring their well-being and active participation in the community. The focuses are 1) housing; 2) community project; 3) local research & perspective; 4) ethnic variations in perceptions of AF; 5) initiatives on social vulnerability. | (Chan et al., 2016; Phillips et al., 2018) |
|  | Taiwan, Republic of China | Many cities adopted the WHO's AFC guides since 2010. | World Health Organization | Local governments, policymakers, urban planners, and researchers. | Adopted and applied the WHO AFC guides to major cities, implementing the AFC framework focus on eight key domains: outdoor spaces, transportation, housing, social participation, respect and inclusion, civic participation, communication, and community support/health services. | (Liu et al., 2018; Chao & Huang, 2016) |
| South America | Countries south of the United States | Adopted and applied the WHO AFC guides. | World Health Organization | Local and national governments, policymakers, and urban planners. | Adopted and applied the 8 domains from the WHO AFC guides. Called for greater policy flexibility to accommodate Latin American social structures, such as extended family caregiving. | (Guillemot & Warner, 2023; Rodriguez-Rodriguez & Sanchez-Gonzalez, 2016). |
|  | Sao Paulo, Brazil | Age-Friendly Sao Paulo program - Elder-Friendly Cities Seal. 2012. | The state of São Paulo | São Paulo policymakers and advocacy groups. | This program is about creating the environment and opportunities that enable older people to be and do what they value throughout their lives  The key areas are: 1) creation of a City Council for the Elderly, 2) complete policy assessments, 3) registration of all low-income older adults in the public services system and at community health centers, and 4) taking any other actions deemed appropriate by the individual city. | (Guillemot & Warner, 2023) |
| Africa | / | / | / |  | / | / |
